# Intravenous Nanocarrier for Improved Efficacy of Quercetin and Curcumin against Breast Cancer Cells: Development and Comparison of Single and Dual Drug–Loaded Formulations Using Hemolysis, Cytotoxicity and Cellular Uptake Studies

**DOI:** 10.3390/membranes12070713

**Published:** 2022-07-15

**Authors:** Mohammad Akhlaquer Rahman, Vineet Mittal, Shadma Wahab, Abdulrhman Alsayari, Abdullatif Bin Muhsinah, Dalia Almaghaslah

**Affiliations:** 1 Department of Pharmaceutics and Industrial Pharmacy, College of Pharmacy, Taif University, Taif 21974, Saudi Arabia; 2 Department of Pharmaceutical Sciences, Maharshi Dayanad University, Rohtak 124001, India; drvineet.pharma@mdurohtak.ac.in; 3 Department of Pharmacognosy, College of Pharmacy, King Khalid University, Abha 61421, Saudi Arabia; sabdulwahab@kku.edu.sa (S.W.); alsayari@kku.edu.sa (A.A.); ajmohsnah@kku.edu.sa (A.B.M.); 4 Complementary and Alternative Medicine Unit, King Khalid University, Abha 61421, Saudi Arabia; 5 Department of Clinical Pharmacy, College of Pharmacy, King Khalid University, Abha 61421, Saudi Arabia; damoazle@kku.edu.sa

**Keywords:** breast cancer, quercetin, curcumin, hemocompatibility, cytotoxicity, cellular uptake

## Abstract

The present work highlights the suitability of an oil-based nanocarrier to deliver quercetin (Q) and curcumin (C) through the intravenous route for treatment of breast cancer. The nanoemulsion prepared by the modified emulsification-solvent evaporation method resulted in particle size (<30 nm), polydispersity index (<0.2), zeta potential (<10 mV), optimum viscosity, high encapsulation efficiency and drug loading for both drugs. The pH and osmolarity of the nanoemulsion were about 7.0 and 280 mOsm, respectively, demonstrated its suitability for intravenous administration. In-vitro release of drugs from all the formulations demonstrated initial fast release followed by sustained release for a period of 48 h. The fabricated single and dual drug–loaded nanoemulsion (QNE, CNE, QC-NE) exhibited moderate hemolysis at a concentration of 50 μg/mL. The % hemolysis caused by all the formulations was similar to their individual components (*p* ˃ 0.05) and demonstrated the biocompatibility of the nanoemulsion with human blood. In vitro cytotoxic potential of single and dual drug–loaded nanoemulsions were determined against breast cancer cells (MF-7). The IC_50_ value for QNE and CNE were found to be 40.2 ± 2.34 µM and 28.12 ± 2.07 µM, respectively. The IC_50_ value for QC-NE was 21.23 ± 2.16 µM and demonstrated the synergistic effect of both the drugs. The internalization of the drug inside MF-7 cells was detected by cellular uptake study. The cellular uptake of QNE and CNE was approximately 3.9-fold higher than free quercetin and curcumin (*p* < 0.0001). This strategically designed nanoemulsion appears to be a promising drug delivery system for the proficient primary preclinical development of quercetin and curcumin as therapeutic modalities for the treatment of breast cancer.

## 1. Introduction

**Figure 1:**
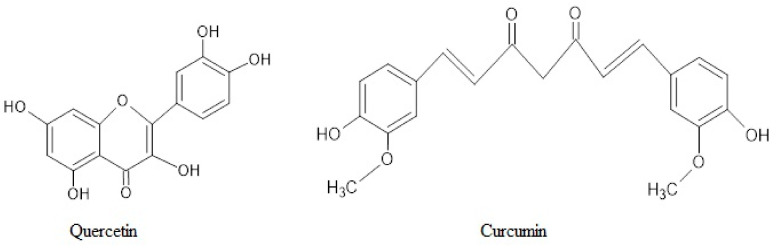
Chemical structure of quercetin (Q) and curcumin (C).

Breast cancer is the most frequent type of cancer in women and reported a high mortality rate [1]. There is considerable evidence supporting the anticancer properties of naturally occurring chemicals that could be effective in a variety of cancer prevention and treatment methods [2,3,4]. Quercetin (Q) and curcumin (C) are among the most popular naturally occurring compounds reported to have anticarcinogenic properties in breast cancer [5,6,7,8]. Quercetin and curcumin (Figure 1) prevent breast cancer by a multiple mechanism, including promoting cancer cell apoptosis and decreasing tumor cell proliferation and metastasis [9,10,11,12].

Despite the potential anticarcinogenic effect, the use of quercetin and curcumin remains limited due to their poor aqueous solubility and absorption in the gastrointestinal tract [13]. Recent studies have shown that nanotechnology-based formulations, including nanoemulsions and many others, can be employed to address a variety of quercetin bioavailability [14,15] and curcumin bioavailability concerns [13,16]. Nanoemulsions are essentially transparent or translucent dispersions of oil and water stabilized by an interfacial coating of surfactant molecules with droplet sizes smaller than 100 nm and demarcated as thermodynamically stable formulations. A nanoemulsion offers high solubilization capacity for poorly soluble drugs and potential for enhanced therapeutic efficacy. Moreover, it offers anticipated patient acceptance and compliance due to reduced side effects [17]. Furthermore, targeting tumors with nanotechnology-based formulations administered via different routes of administration may boost chemopreventive and chemotherapeutic effects. Parenteral approaches are usually employed in early development of drugs for preclinical studies, including pharmacokinetics, pharmacodynamics, and toxicity studies [18,19]. Among all the parenteral routes, the intravenous route is the most efficient but most restrictive in terms of requirements [18]. According to the pharmacopeia and reports in the literature, intravenous formulations need to be sterile, particulate-free, isotonic, preferably between pH 2 and 9 and their hemolytic potential must be assessed [19]. Indeed, red blood cell (RBCs) lysis by its formulation components is a well-known phenomenon [20,21]. When the hemolytic potential of the excipients used in intravenously delivered formulations is extremely high, it might result in anemia and even death [22]. The biocompatibility of each excipient and nanoformulation with blood components appears to be crucial in early preclinical development. The selection of excipients can be adjusted at the formulation development stage by understanding their erythrocyte-damaging potential. To be more predictive and to mimic the in vivo conditions, this test has to be performed with human blood [19,23]. Examining their cytotoxic potential is the second stage in the development of anticarcinogenic drugs. In vitro anticarcinogenic potential is usually determined on human breast cancer cell lines (MF-7 cells). Understanding the connection between nanocarrier uptake and its efficacy for cancer treatment is also important in early drug development. In this perspective, the cellular uptake of the nanocarrier by cancer cells is expected to enhance the treatment efficacy. When drugs are combined in a single nanocarrier, combination therapy is able to overcome resistance as well as cross resistance, which is frequently linked with traditional chemotherapy. Hence, the goal of this study is to create a safe, repurposed drug-loaded nanoemulsion with better pharmacokinetics and therapeutic efficacy for breast cancer treatment.

## 2. Materials and Methods

### 2.1. Materials

Quercetin (MW: 320.23 g/mol) and curcumin (MW: 368.38 g/mol) were obtained from Loba chemicals, Bangalore, India as free gift sample. Kamani Oil Industries Ltd., Mumbai, India provided the gift sample of soy lecithin. Sefsol oil was a kind gift sample from Nikko Chemicals, Tokyo, Japan. Polysorbate 80 was purchased from Sigma Aldrich, USA. Human breast cancer cells (MF-7 cells) were purchased from the American Culture Collection, USA. Other chemicals used were in the grade of analytical reagent category.

### 2.2. Preparation of Single and Dual Drug–Loaded Nanoemulsion

Nanoemulsion was prepared by modified emulsification-solvent evaporation method [24,25]. The aqueous phase was prepared by dissolving polysorbate 80 in double-distilled water. The isotonicity of aqueous phase was adjusted with 2% (*w*/*v*) glycerol. For the preparation of oil phase, soy lecithin was dissolved into a mixture of acetone: ethanol (50:50, *v*/*v*) in one beaker and in another, drug was dispersed into sefsol oil (0.05% *w*/*w*). Contents of both the beakers were mixed together resulting in formation of oil phase. The obtained oil phase was immediately added to aqueous phase with moderate stirring followed by sonication for 5 min, which leads to the formation of nanoemulsion. The solvent and excess water were removed by keeping the nanoemulsion under reduced pressure. The final concentration of nanoemulsion was 15% *v*/*v* of initial volume from the aqueous phase. The amount of polysorbate 80 and soy lecithin was fixed at 3.5% and 4% (*w*/*w*), respectively. The final drug content in the nanoemulsion was 10 mg/mL and was adjusted between pH 6.5–7.5 with 0.1 N NaOH solution. The binary mixtures (QC-NE) were prepared taking quercetin and curcumin in a 1:1 proportion by weight.

### 2.3. Droplet Size, Polydispersity Index and Zeta Potential Measurement

Dynamic light scattering technique was used to determine the average droplet size and polydispersity index of nanoemulsion. The apparatus used for the purpose was NanoZS^®^ (Malvern Instruments, Worcestershire, UK) equipped with a 633 nm laser at a fixed scattering angle of 173°. The samples were diluted with 1 mM NaCl (1:100) to ensure the suitable angle of scattering on the detector. The electrophoretic mobility of nanoemulsion droplets was used to calculate the zeta potential using Smoluchowski equation [26]. Temperature of cells was kept constant at 25 °C. Other parameters were set to: dielectric constant, 7.6; refractive index, 1.33; and a cell voltage of 40 kV.

### 2.4. Viscosity Measurement

Undiluted nanoemulsion (6 mL) was used as sample to determine the viscosity using Brookfield Digital Rheometer (Model DVIII, Brookfield Engineering Laboratories Inc., Middleboro, MA, USA). The viscometer was assembled with SC4-18 spindle. The reading was taken at a fixed temperature of 25 °C and the values were analyzed using Rheocalc V3.1-1 software.

### 2.5. pH and Osmolarity

A pH meter (Eutech instrument, Landsmeer, The Netherlands) equipped with a microprobe (Fisher Scientific, France) was utilized to measure the pH of the nanoemulsion; 5 mL nanoemulsion was taken in a glass beaker and microprobe was dipped into the sample to assess the reading. The freezing point method was used to assess the osmolarity of the nanoemulsion; 100 µL sample was applied in the microtube of a micro-osmometer instrument autocal type 15/15M (Loser Messtechnik, Berlin, Germany) and the measurements were executed.

### 2.6. Determination of Encapsulation Efficiency 

The formulation was filtered through 0.2 µm syringe filters (Sartorius, Goettingen, Germany). The filtered sample was diluted with methanol (1:500, *v*/*v*) and the concentration of quercetin and curcumin was determined using UV spectrophotometer (UV 1700, Shimadzu Corporation, Kyoto, Japan) at a wavelength of 380 nm and 430 nm, respectively. The purpose of filtration was to remove the unentrapped drugs. The encapsulation efficiency (EE) and drug loading (DL) were calculated using the following equation:

(1) $$\mathrm{Encapsulation}\;\mathrm{efficiency}\;(\%\;\mathrm{EE})=\frac{\mathrm{Quantity}\;\mathrm{of}\;\mathrm{drug}\;\mathrm{entrapped}}{\mathrm{Total}\;\mathrm{quantity}\;\mathrm{of}\;\mathrm{drug}\;\mathrm{added}\;}\times 100$$

(2) $$\mathrm{Drug}\;\mathrm{loading}\;(\%\;\mathrm{DL})=\frac{\mathrm{Quantity}\;\mathrm{of}\;\mathrm{drug}\;\mathrm{entrappesd}}{\mathrm{Total}\;\mathrm{quantity}\;\mathrm{of}\;\mathrm{lipid}\;\mathrm{excipients}\;\mathrm{added}}\times 100$$

### 2.7. In Vitro Drug Release Study

In vitro release study of quercetin and curcumin from all the formulations were performed using dialysis bag method; 0.5 mL of each formulation was separately filled into the dialysis bag (MWCO: 12,000 g/mol). The dialysis bag was then dipped into a beaker containing 50 mL phosphate buffer solution (pH 7.4). The temperature of dissolution media was maintained at 37 ± 0.5 °C with mild agitation (50 rpm), and 1 mL of sample was withdrawn at pre-determined time interval (0, 1, 2, 4, 6, 12, 24 and 48 h), and the same volume was replaced with fresh dissolution media to maintain sink condition. The samples were analyzed for quercetin and curcumin content using UV spectrophotometer (UV 1700, Shimadzu Corporation) at a wavelength of 380 nm and 430 nm, respectively. Finally, the percentage cumulative release of drug was determined and a graph was plotted between % cumulative drug release vs. time.

### 2.8. Hemolytic Potential of Nanoemulsion and Individual Components

Hemolysis test was performed using human blood taken from healthy volunteer after signing a written consent. The whole blood was defibrinogenated before further study and was achieved by mixing blood with glass beads in an airtight container at room temperature for not more than 1 h; 10 mL of defibrinogenated blood was used to obtain the erythrocyte pellets (RBCs). The RBCs pellets were diluted with 0.9% NaCl solution to get 3% final dispersion and kept in a refrigerator for further use. The Q-NE, CNE, QC-NE and its individual components (Soy lecithin, polysorbate 80) were then added to 1 mL of above dispersion. Then, the volume was diluted to 5 mL with 0.9% NaCl solution to obtain 50 μg/mL of free drug, the formulation and its individual components. The above samples were then incubated for 2 h at 37 °C. After 2 h of incubation and were centrifuged using REMI R4C centrifuge (Mumbai, India) at 2000 rpm for 10 min. The clear supernatant was subjected for absorbance measurement using a UV-visible spectrophotometer at a wavelength of 570 nm. The de-ionized water was used as positive (+ve) control and 0.9% NaCl solution as negative (−ve) control. The negative control was prepared by diluting 100 µL of cell suspension with 3 mL with 0.9% NaCl solution. The positive control was prepared by adding 100 µL of cell suspension to 3 mL de-ionized water. The % hemolysis was calculated using the equation mentioned below [27,28].

(3) $$Hemolysis\;\left(\%\right)=\frac{\mathrm{Abs}.\;\mathrm{of}\;\mathrm{test}\;\mathrm{sample}-\mathrm{Abs}.\;\mathrm{of}\;\left(-\mathrm{ve}\right)\;\mathrm{control}}{\mathrm{Abs}.\;\mathrm{of}\;\left(+\mathrm{ve}\right)\;\mathrm{control}-\mathrm{Abs}.\;\mathrm{of}\;\left(-\mathrm{ve}\right)\;\mathrm{control}\;}\times 100$$

### 2.9. In Vitro Cytotoxicity Study Using MCF-7 Cells

For the culture of cells, one sachet of Dulbecco’s Modified Eagle’s Medium (DMEM) was dissolved in Milli-Q water and the pH was adjusted to 7.4 with 1 N HCl. The contents of one sachet were dissolved in Milli-Q water. The pH was maintained to 7.4 with 1 N HCl and the volume was made up to 1 L with Milli-Q water. Above prepared media were then filtered through 0.2 μm membrane filter under aseptic conditions. A stock solution (5 mg/mL) was prepared by adding MTT [3-(4,5-dimethylthiazol-2-yl)-2,5-diphenyltetrazolium bromide] reagent in DMEM medium and filtered again using 0.2 μm membrane filter. Breast cancer cells (MCF-7 cells) were seeded in a 96-well plate at a concentration of 100 cells/µL of medium/well. The plates were incubated at 37 °C temperature in the humidified atmosphere of CO_2_ (5%) and allowed to grow for 24 h. After 24 h of incubation, different concentrations of QNE, CNE and QC-NE ranging from 2.5 µM–160 µM was applied on MCF-7 cells plated into 96-well plates. Untreated cells containing only the media were used as control, and 20 µL of MTT solution (5 mg/mL) was added to each well and kept in an incubator for 2 h. The medium from each well was then aspired, and 200 µL of dimethyl sulfoxide (DMSO) was added to the wells, mixed thoroughly and left at room temperature for 10 min. The absorbance of DMSO extract was measured at 590 nm using an ELISA plate reader. The death at minimum and maximum concentration and IC_50_ was compared for all the samples. 

### 2.10. In Vitro Cellular Uptake Study Using MCF-7 Cells

The breast cancer cells (MCF-7) were used for cellular uptake of QNE, CNE and QC-NE. Briefly, 3 mL of 1 × 10^6^ cells/well were placed into 6-well plates and incubated at 37 °C for 24 h. After 24 h of incubation, the cells were exposed with each formulation (QNE, CNE and QC-NE) at a concentration of 20 μg/mL, followed by incubation at 37 °C for 4 h. The wells were washed with phosphate-buffered saline (PBS). Lysis of cells was achieved using 0.5 mL double distilled water/well, and then 0.5 mL acetonitrile was added in each well to extract the drug. The purpose of adding acetonitrile was to precipitate out proteins from the lysed cells. The whole content was centrifuged at 15,000 rpm for 5 min. The clear supernatant was filtered through a 0.45 μm filter before analysis. The samples were injected into the HPLC column at the rate of 1 mL/min. 

### 2.11. HPLC Method for Analysis of Quercetin and Curcumin

The instrument (Shimadzu LC-10AT VP) was equipped with C18 column (25 cm × 4.6 mm, 5 mm particle size) protected by a C18 guard column and used for analysis of samples. The optimized mobile phase was aqueous phosphoric acid 1% *w*/*v* adjusted at pH 2.6 (Eluent A) and acetonitrile (Eluent B) at a flow rate of 1.0 mL/min, operated in gradient elution mode. The mobile phase was selected on the basis of sharp peak and low retention time of each analyte. Temperature of the column was set to 40 °C. The detection was achieved using UV detector (Shimadzu, Kyoto, Japan) at a wavelength of 400 nm. Class VP, version 5.032 software was used for acquisition of data. 

### 2.12. Stability Study

Monitoring changes in the nanoemulsion droplet size, polydispersity index, zeta potential, osmolarity and pH under standard storage conditions (4 °C) allowed researchers to determine its stability. Additionally, stability tests were carried out on samples kept at 25 °C temperature. Testing was done after intervals of 0 h, 1, 3 and 6 months.

### 2.13. Statistical Analysis

Statistical analysis was carried out using one-way ANOVA with the help of Graph Pad Prism.

## 3. Results and Discussion

### 3.1. Physicochemical Characteristics of Intravenous Nanoemulsion

The formation of a nanoemulsion generally requires energy input and its physicochemical properties depend on their composition, method of preparation and order of component addition. Moreover, the in vivo performance of the final product depends on the physicochemical characteristics of the nanoemulsion. The physicochemical characteristics of nanoemulsions are given in Table 1. In our study, polysorbate 80 was used as a hydrophilic emulsifier and soy lecithin as a lipophilic emulsifier in the development of nanoemulsions. Polysorbate 80 is a synthetic nonionic surfactant commonly used as excipient of choice as a solubilizing, stabilizing, and emulsifying agent in formulation development. In the past few decades, several polysorbate 80–based drug products emerged as a therapeutic delivery system in the oncology setting for chemotherapy, supportive care or prevention. The nanoemulsions prepared in our study were prepared with the aim of being safe and compatible with the blood. Soy lecithin reported to appear quite safe regarding hemocompatibility [29]. Polysorbate 80 and soy lecithin help in formation of the aqueous and lipid phase, respectively. Nanoemulsions could be formed spontaneously after the organic solvent was removed from the emulsions. The droplet size of the nanoemulsion was in the nanoscale range. The polydispersity index demonstrated its narrow size distribution. The biodistribution of the nanocarrier may be affected by their surface charge. The zeta potential with negative charge ensures good electrochemical stability of the nanoemulsion compared to the neutral surface [30,31]. Neutral nano-droplets (±10 mV) display diminished phagocytic uptake compared to positive- or negative-charged particles [32], which is favorable to achieve efficient drug delivery after intravenous administration. The zeta potential value observed designates its stability towards aggregation. The results of viscosity showed that there was no significant difference in viscosity values between the nanoemulsions. All of data showed that the rheology of the nanoemulsion followed Newtonian principles [33]. The pH and osmolarity of the parenteral formulation must be adjusted for safe delivery of the administered dose. The pH and osmolarity of the nanoemulsion established its suitability for intravenous administration. All the nanoemulsion formulations demonstrated high encapsulation efficiency and drug loading for both the drugs, advocating the lipophilic nature of quercetin and curcumin.

### 3.2. In Vitro Drug Release Study

**Figure 2:**
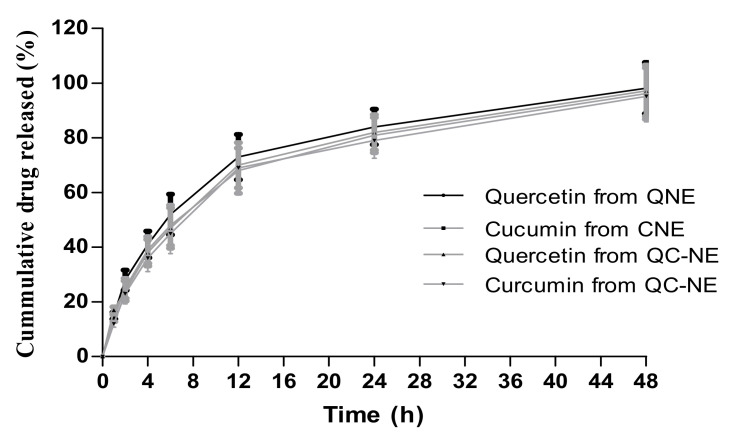
Percentage cumulative release of quercetin and curcumin form nanoemulsion formulations at different time intervals.

The release behaviors of quercetin and curcumin form nanoemulsion formulations (QNE, CNE and QC-NE) in phosphate buffer (pH 7.4) were observed using the dialysis membrane method (Figure 2). All the nanoemulsion formulations demonstrated a cumulative release ˃ 95% in 48 h. There was no significant difference between the formulations in terms of percentage cumulative release at specific time points. The initial fast release can be attributed to the fact that the small size of the nanoemulsion droplet provides larger surface area for the drug to be released. Additionally, less time is required to release the drug from outer core of the nanoemulsion when it is near the dissolution media. Thus, a faster rate of drug release can be observed in the initial 2 h and sustained release afterward for a period of 48 h.

### 3.3. In Vitro Hemolysis of Free Drug, Nanoemulsion and Its Individual Components

**Figure 3:**
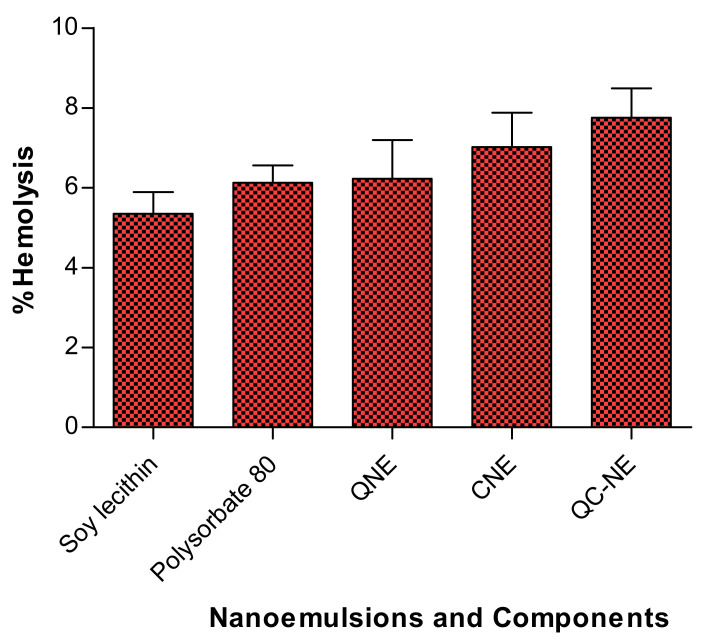
Hemolysis (%) of soy lecithin, polysorbate 80, QNE, CNE and QC-NE. *p* > 0.05.

The toxicity of nanomedicine is a key issue in the way of drug design. The nanomedicine must be biocompatible with blood components. There are only a few approved excipients available for parenteral administration. Hence, utmost care must be taken in the drug designing process, especially for parenteral delivery. The acceptability of the nanoemulsion was verified by the assessment of the hemolysis of RBCs (results shown in Table 2 and Figure 3). Double-distilled water was used as +ve control and showed 100% hemolysis of RBCs. As no hemolysis (0%) was observed with 0.9% NaCl, it was used as –ve control. The % hemolysis values of QNE, CNE and QC-NE were 6.23 ± 0.97%, 7.02 ± 0.86% and 7.76 ± 0.73%, respectively. The % hemolysis values of soy lecithin and polysorbate 80 were 5.35 ± 0.54% and 6.13 ± 0.43%, respectively. The QNE, CNE and QC-NE exhibited moderate hemolysis at a concentration of 50 μg/mL and was similar to their individual components (*p* > 0.05)**.** The results demonstrated that the nanoemulsion components are biocompatible. Moreover, inclusion of all the components into nanoemulsion changes their degree of interaction with the erythrocytes but does not show any additive effect on the hemolysis. The % of hemolysis induced by nanoemulsion remained within the acceptable range for parenteral administration [34].

### 3.4. In Vitro Cytotoxic Effect of Drug from Single and Dual Drug–Loaded Nanoemulsion

**Figure 4:**
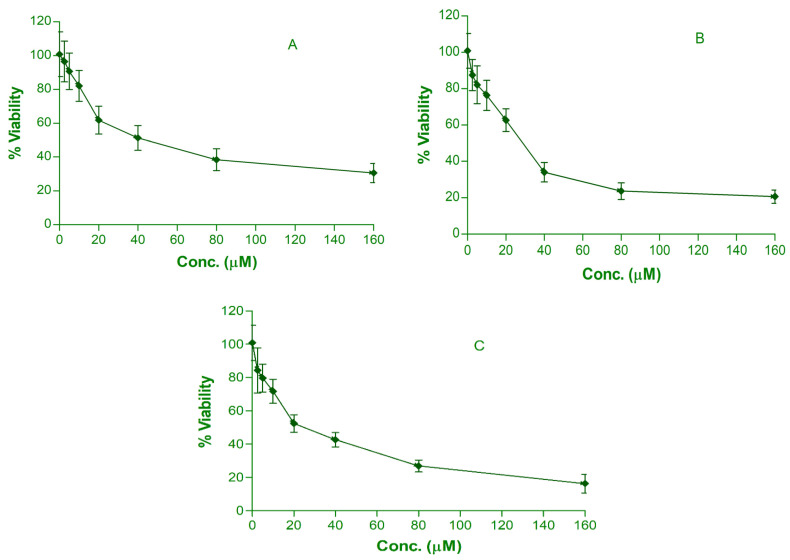
Viability (%) of QNE- (**A**), CNE- (**B**) and QC-NE (**C**)-treated MCF-7 cells. IC_50_ values for QNE-, CNE- and QC-NE-treated MCF-7 cells were identified to be 40.2 µM, 28.12 µM and 21.23 µM, respectively.

**Figure 5:**
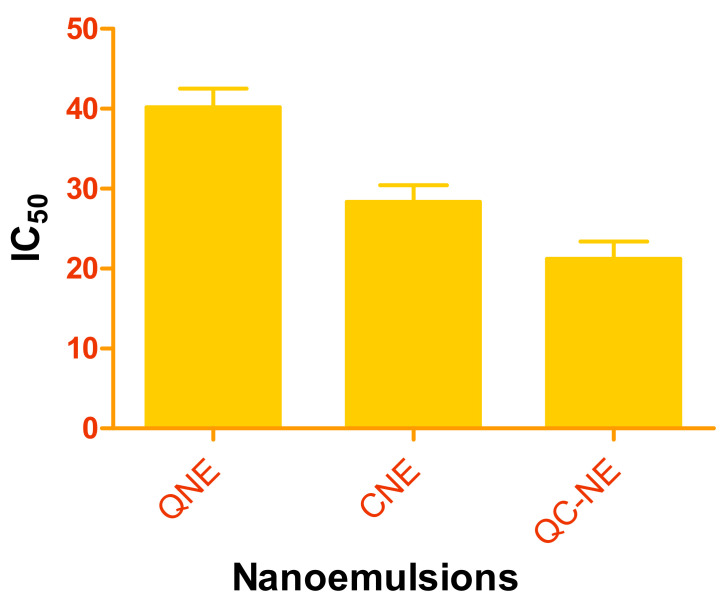
IC_50_ value of nanoemulsions (QNE, CNE and QC-NE).

The classical chemotherapeutics used in cancer therapy have many side effects. Moreover, they exhibit a high level of toxicity in patients. Drug design using nanotechnology may imply the uses of phytochemicals, and ultimately, the nanoscale product may offer low toxicity and fewer side effects. Furthermore, the nanoscale product may prove more effective in low doses than the normal-sized agents against cancer cells. The treatment with nanoparticles led to the inhibition of the growth of MCF-7 cells. It was observed that after 24 h of MTT assay, both quercetin and curcumin were cytotoxic, showing linear relationships between the concentrations of drugs vs % viability. The results of the cytotoxicity study are demonstrated in Table 2 and Figure 4. Figure 5 represents the IC_50_ value of all the developed nanoemulsions. The maximum and minimum % viability for free quercetin were 96.54 ± 12.05% and 30.56 ± 5.62% at concentrations of 2.5 µM and 160 µM, respectively. The IC_50_ value was found to be 40.2 ± 2.34 µM. The CNE demonstrated maximum % viability 87.44 ± 10.15% and minimum % viability 20.48 ± 3.43% at curcumin concentrations of 160 μM and 2.5 µM, respectively. The IC_50_ value was found to be 28.12 ± 2.07 µM. Co-loading of quercetin and curcumin in the nanoemulsion demonstrated a synergistic effect against breast cancer cells. The maximum and minimum % viability were 84.33 ± 8.26% and 16.27 ± 2.87% at 2.5 µM and 160 μM concentrations for both quercetin and curcumin, respectively, when used in combination. The IC_50_ value was observed to be 21.23 ± 2.16 µM, demonstrating the synergistic effect of both the drug. QC-NE exhibited the maximum cytotoxicity against breast cancer cells and was hence the most effective among all the formulations. 

### 3.5. Cellular Uptake of Drug from Single and Dual Drug–Loaded Nanoemulsion 

**Figure 6:**
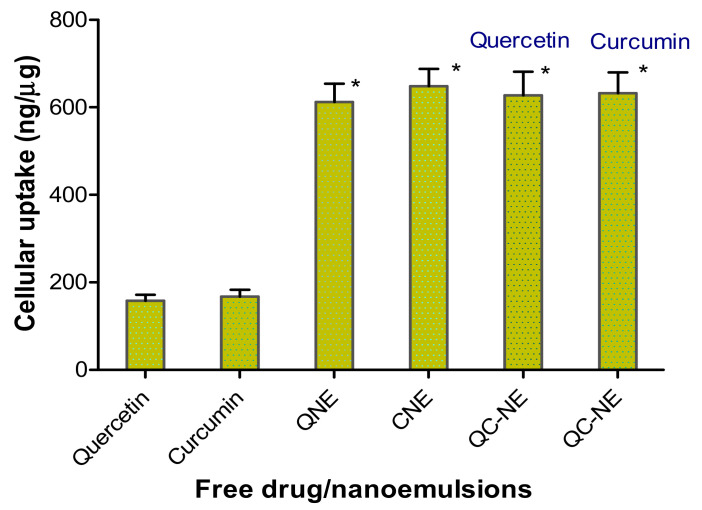
Cellular uptake (ng/µg) of free quercetin, curcumin and nanoemulsions (QNE, CNE and QC-NE). * *p* < 0.0001 versus free drug.

The cellular uptake of quercetin and curcumin into breast cancer cells was used to investigate the effect of the nanoemulsion formulation on augmenting uptake of both the drugs into an in vitro model of breast cancer cells (MCF-7 cells). Cellular uptake (ng/µg) of free quercetin, curcumin and nanoemulsions (QNE, CNE and QC-NE) are shown in Figure 6. The uptake of free quercetin and curcumin were found to be 158.3 ± 13.5 ng/μg and 167.5 ± 15.8 ng/μg, respectively. The QNE and CNE demonstrated cellular uptake of 612.3 ± 41.4 ng/μg and 648.6 ± 39.6 ng/μg, respectively. The cellular uptake of QNE and CNE was approximately 3.9-fold higher than free quercetin and curcumin. The increased cellular uptake of QNE and CNE was attributed to high internalization of the nanocarrier system mainly due to presence of lipids as one of the major components in drug development. The cellular uptake of free quercetin and curcumin was significantly different compared to the uptake of the nanoemulsion formulation. There was no significant difference in the cellular uptake of the three nanoemulsion formulations.

### 3.6. Stability Study

The intravenous nanoemulsions are commonly stored at refrigerated temperature. Hence, for the stability study, the formulations were kept in a refrigerator maintained at a temperature of 4 °C for a period of 6 months and assessed for various parameters. The established stability data suggested that the developed nanoemulsions were stable in nature (Table 3). The pH value for nanoemulsions kept at refrigerated temperature were within the requirements for intravenous formulation in which it should ideally be neutral, but can reside within the range of 4–9 [35]. Over a storage period of 6 months, no significant differences (*p* > 0.05) in droplet size, polydispersity index, zeta potential, osmolarity and pH values of nanoemulsions were noticed. On the other hand, the stability of nanoemulsions at elevated temperature (25 °C) showed no significant difference in droplet size, polydispersity index, zeta potential and osmolarity values but a slight decrease in pH values was observed. The stability data obtained from both the storage conditions showed that the physical character of the drug-loaded nanoemulsions did not undergo any significant change during 6 months of storage. However, the slight change in pH of the nanoemulsions was observed at 25 °C might be due to the hydrolysis of the non-aqueous components of the nanoemulsion. Moreover, no significant difference in drug content over the period of 6 months was noticed at both the storage conditions (4 °C and 25 °C). 

## 4. Conclusions

In our study, three formulations containing single and dual drug–loaded nanoemulsions were successfully prepared using the proposed method. The nanoemulsion generated from a simple modified emulsification-solvent evaporation method appeared very performant to improve solubility of practically insoluble drugs such as quercetin and curcumin. The prepared nanoemulsion formulations were spherical in shape, demonstrated uniform size distribution, high encapsulation efficiency and drug loading. In addition, cell culture studies proposed that encapsulation of quercetin and curcumin within the nanoemulsion formulations proficiently improved the cellular uptake of both the drugs by breast cancer cells and exerted a remarkable cytotoxic effect on breast cancer cells. Moreover, the dual drug–loaded nanoemulsions demonstrated a synergistic cytotoxic effect on breast cancer cells. The stability studies indicated good stability of developed nanoemulsions for 6 months when stored at 4 °C based on droplet size, polydispersity index, zeta potential and pH values. No changes in drug concentrations were observed after 6 months of storage. Overall, the study revealed the potential application of quercetin and curcumin in single as well as in combination formulations against breast cancer. However, further in vivo studies are needed to validate these results.

## Figures and Tables

**Table 1:** Characteristics of nanocarrier systems meant for intravenous administration.

Nanocarriers	Droplet Size (nm)	Polydispersity Index	Zeta Potential (−mV)	Viscosity (cps)	pH	Osmolarity (mOsm)	% EE	% DL
QNE	26.4 ± 1.34	0.126 ± 0.002	8.6 ± 1.62	1.64	7.1 ± 0.2	279 ± 8.67	90.28	0.73
CNE	27.8 ± 1.87	0.129 ± 0.001	8.4 ± 1.38	1.76	7.2 ± 0.1	279 ± 7.89	87.54	0.81
QC-NE	25.9 ± 1.59	0.127 ± 0.003	8.9 ± 1.57	1.69	7.0 ± 0.3	280 ± 9.36	Q: 88.83	Q: 0.71
							C: 85.37	C: 0.83

**Table 2:** Biological evaluation of various samples meant for intravenous administration.

Test Sample	% Hemolysis	In Vitro Cytotoxicity			In Vitro Cellular Uptake (ng/μg)
		Max. Viability (%)	Min. Viability (%)	IC_50_ (µM)	
Quercetin	-	-	-	-	158.3 ± 13.5
Curcumin	-	-	-	-	167.5 ± 15.8
Soy lecithin	5.35 ± 0.54	-	-	-	-
Polysorbate 80	6.13 ± 0.43	-	-	-	-
QNE	6.23 ± 0.97	96.54 ± 12.05	30.67 ± 5.62	40.2 ± 2.34	612.3 ± 41.4
CNE	7.02 ± 0.86	87.44 ± 10.15	20.48 ± 3.43	28.12 ± 2.07	648.6 ± 39.6
QC-NE	7.76 ± 0.73	84.33 ± 8.26	16.27 ± 2.87	21.23 ± 2.16	Q:627.5 ± 53.8
					C:632.4 ± 47.6

**Table 3:** Effect of storage condition and time on droplet size, polydispersity index, zeta potential, osmolarity and pH value.

Nanoemulsions	Time (Month)	Droplet Size (nm) at 4 °C and 25 °C	Polydispersity Index at 4 °C and 25 °C	Zeta Potential (−mV) at 4 °C and 25 °C	Osmolarity (mOsm) at 4 °C and 25 °C	pH at 4 °C and 25 °C
QNE	0	26.4 ± 1.34	0.126 ± 0.002	8.6 ± 1.62	279 ± 8.67	7.1 ± 0.2
	1	26.5 ± 1.28	0.126 ± 0.004	8.3 ± 1.34	279 ± 7.05	7.0 ± 0.1
		26.5 ± 1.33	0.126 ± 0.002	8.3 ± 1.27	279 ± 8.35	6.8 ± 0.2
	3	26.5 ± 1.31	0.125 ± 0.001	8.2 ± 1.42	279 ± 6.83	6.9 ± 0.1
		26.6 ± 1.28	0.125 ± 0.003	8.1 ± 1.36	280 ± 5.76	6.5 ± 0.1
	6	26.6 ± 1.24	0.125 ± 0.003	8.2 ± 1.48	280 ± 5.97	6.9 ± 0.3
		26.6 ± 1.18	0.124 ± 0.002	8.0 ± 1.29	280 ± 6.44	6.3 ± 0.3
CNE	0	27.8 ± 1.87	0.129 ± 0.001	8.4 ± 1.38	279 ± 7.89	7.2 ± 0.2
	1	28.0 ± 1.65	0.129 ± 0.002	8.4 ± 1.13	280 ± 6.54	7.2 ± 0.1
		28.2 ± 1.38	0.129 ± 0.003	8.4 ± 1.24	279 ± 7.38	7.2 ± 0.2
	3	28.3 ± 1.41	0.132 ± 0.004	8.6 ± 1.32	280 ± 5.76	7.0 ± 0.1
		28.5 ± 1.39	0.130 ± 0.003	8.7 ± 1.41	279 ± 6.38	6.7 ± 0.3
	6	28.5 ± 1.33	0.131 ± 0.002	8.6 ± 1.42	281 ± 7.04	7.0 ± 0.2
		28.9 ± 1.54	0.130 ± 0.003	8.4 ± 1.29	280 ± 6.46	6.4 ± 0.2
QC-NE	0	25.9 ± 1.59	0.127 ± 0.003	8.9 ± 1.57	280 ± 9.36	7.0 ± 0.2
25.9 ± 1.59	1	25.9 ± 1.46	0.127 ± 0.002	8.9 ± 1.82	280 ± 5.89	7.2 ± 0.3
		25.7 ± 1.37	0.128 ± 0.003	8.7 ± 1.46	280 ± 7.28	6.9 ± 0.2
	3	26.3 ± 1.53	0.128 ± 0.004	8.7 ± 1.37	279 ± 8.74	7.2 ± 0.1
		25.1 ± 1.48	0.129 ± 0.001	8.4 ± 1.29	279 ± 7.46	6.6 ± 0.2
	6	26.5 ± 1.52	0.128 ± 0.002	8.5 ± 1.64	279 ± 7.03	7.3 ± 0.2
		24.8 ± 1.27	0.130 ± 0.003	8.4 ± 1.72	278 ± 8.16	6.3 ± 0.1

## Data Availability

The data presented in this study are available on request from the corresponding author.
